# Antimicrobial stewardship in long term care facilities: what is effective?

**DOI:** 10.1186/2047-2994-3-6

**Published:** 2014-02-12

**Authors:** Lindsay E Nicolle

**Affiliations:** 1 Health Sciences Centre, University of Manitoba, Room GG443, 820 Sherbrook Street, Winnipeg R3A 1R9, Canada

**Keywords:** Long term care facility, Antimicrobial stewardship, Pneumonia, Urinary tract infection

## Abstract

Intense antimicrobial use in long term care facilities promotes the emergence and persistence of antimicrobial resistant organisms and leads to adverse effects such as *C. difficile* colitis. Guidelines recommend development of antimicrobial stewardship programs for these facilities to promote optimal antimicrobial use. However, the effectiveness of these programs or the contribution of any specific program component is not known. For this review, publications describing evaluation of antimicrobial stewardship programs for long term care facilities were identified through a systematic literature search. Interventions included education, guidelines development, feedback to practitioners, and infectious disease consultation. The studies reviewed varied in types of facilities, interventions used, implementation, and evaluation. Comprehensive programs addressing all infections were reported to have improved antimicrobial use for at least some outcomes. Targeted programs for treatment of pneumonia were minimally effective, and only for indicators of uncertain relevance for stewardship. Programs focusing on specific aspects of treatment of urinary infection – limiting treatment of asymptomatic bacteriuria or prophylaxis of urinary infection – were reported to be effective. There were no reports of cost-effectiveness, and the sustainability of most of the programs is unclear. There is a need for further evaluation to characterize effective antimicrobial stewardship for long term care facilities.

## Review

### Introduction

There is intense antimicrobial use in long term care facilities. A recent systematic review reported 47% to 79% of nursing home residents receive systemic antimicrobials each year, and antimicrobials are prescribed for 77 to 88% of all infectious episodes [1]. The prevalence of antimicrobial use varies from 4.8 – 15.2%. There is also substantial variation in use among facilities, with rates from 0.4 – 23.5/1,000 resident days reported for US facilities [2]. Reviews have consistently concluded that a high proportion of this antimicrobial use is inappropriate [3]. The intense antimicrobial pressure in these settings leads to a number of adverse effects including adverse drug effects and *Clostridium difficile* colitis [4]. A consequence of particular concern is the emergence and persistence of antimicrobial resistant bacteria in residents of these facilities. Studies reported globally identify a high and increasing prevalence of antimicrobial resistance in long term care facilities, with the type and amount of antimicrobial exposure consistently correlated with antimicrobial resistance [1].

There are many challenges to address in improving antimicrobial use in long term care facilities. Residents in these facilities have a high incidence of infection because of underlying comorbidities, use of invasive devices, aging associated changes, and institutional exposure [3]. There is often diagnostic uncertainty because of limitations in the clinical and microbiological evaluation. With the high prevalence of asymptomatic bacteriuria, of oropharyngyal colonization with gram negative organisms, and of colonization of skin sites such as feeding tubes or decubitus ulcers with potentially pathogenic organisms, bacterial cultures are frequently positive in the absence of infection [3]. Thus, microbiologic tests must be interpreted critically in the context of the clinical presentation. Antimicrobial therapy is frequently prescribed for non-specific clinical alterations attributed to infection when evidence to confirm infection is not present [5]. This leads to overdiagnosis of some infections and overtreatment with empiric antimicrobial therapy.

Antimicrobial stewardship programs have been recommended to promote optimal antimicrobial use in these settings [3]. The SHEA/APIC guideline for infection prevention in long term care has two recommendations relevant to antimicrobial stewardship [3]. These are, 1: infection control programs in long term care facilities should be encouraged to include a component of antimicrobial stewardship and, 2: the infection control practitioner should monitor antibiotic susceptibility results from cultures to detect clinically significant antibiotic resistant bacteria in the institution, and antibiotic susceptibility trends should be communicated to appropriate individuals and committees. There is, however, no consensus on the specific components of stewardship programs or acknowledgement of resources which need to be applied to support the program. Implementation is hampered by limited resources in these facilities, as well as limited evidence characterizing the effectiveness of a stewardship program or of individual components of such a program. This manuscript reviews current evidence evaluating the effectiveness of antimicrobial stewardship interventions in long term care facilities, summarizes potential effective approaches, and identifies issues which must be addressed to support the development of effective programs in these facilities.

### Methods

Studies published in English between January 1, 2001 and January 1, 2013 describing implementation and evaluation of antimicrobial stewardship programs in long term care facilities were identified through Medline and EMBASE. Key words were (antimicrobial stewardship or use or control) and (long term care facilities or nursing homes). Review articles describing antimicrobial use or stewardship in long term care facilities were also identified and references from these articles scanned for any additional relevant publications. Titles and abstracts of all publications identified were reviewed to select reports appropriate for inclusion. Of the articles identified, 195 were excluded because they were not relevant to antimicrobial stewardship or did not evaluate stewardship interventions.

The studies were considered comprehensive when the antimicrobial stewardship program addressed antimicrobial use for all or most infections, and specific when interventions were targeted to address antimicrobial use for a single type of infection. Program components described in the published reports were identified, and a narrative review summarized outcomes and program characteristics potentially contributing to successful outcomes.

### Results

Four studies describe comprehensive antimicrobial stewardship programs for long term care facilities (Table 1) [6-9]. Two of these reports were cluster randomized controlled trials with multiple facilities participating, and two describe a pre/post analysis of interventions at a single facility. All of the studies reported significant improvements in at least some outcomes following implementation of the programs.

**Table 1:** Reports evaluating the implementation of comprehensive antimicrobial stewardship programs for long term care facilities (LTCF)

**Reference, country**	**Study design**	**Interventions**	**Outcomes**
Schwartz et al., 2007 [6] US	Prospective, before/after; single centre, hospital-based LTC wards; on-site ID consultation.	1. Four teaching sessions over 18 months including all 20 full time staff internists; groups of 3–7.	Pre/post analysis of 100 random charts pre intervention and during 5 months after the last session:
		2. Published guidelines on LTC infections and results of local audit discussed; interactive discussion of local cases.	1. Antimicrobial courses met guideline for diagnostic criteria: 32% vs 62%, p = 0.006
		3. Evidence-based algorithms and guidelines developed with internists.	2. Initial antimicrobial therapy met guidelines: 11% vs 39%; p < 0.001
		4. Pocket booklet with optimal management of LTC infection syndromes.	3. Antimicrobial days fell 29.7%, starts fell 25.9% - improvements sustained 2 yr post-intervention
Monette et al., 2007 [7] Canada	Cluster, randomized controlled trial; 8 LTC, Montreal	Interventions for experimental group:	Experimental vs control homes at trial end:
		1. Mailing antibiotic guide and individual prescribing profile past 3 months to 36 physicians. Antibiotic courses given by physician characterized as adherent or non-adherent.	1. Nonadherent prescriptions: 20.5% vs 5.1%
			2. Likelihood of prescription of nonadherent antibiotics:
			→post-intervention one: OR 0.47, (95% CI 0.21-1.0 1.05)
		2. Repeat second mailing 4 months later.	
			→post-intervention two: OR 0.36 (0.18, 0.73)
			→15 months follow-up: OR 0.48 (0.23-1.02)
Pettersson et al., 2011 [8] Sweden	Cluster, randomized controlled trial; 58 NH	1. Local physician, nurse, developed guidelines in focus groups. Evaluation of guidelines in pilot study with revision.	Effect of intervention (95% CI) at 2 years (differences):
			Primary outcome:
			Fluoroquinolones for UTI: 0.028 (−0.193, 0.249)
		2. Small educational sessions – physicians, nurses.	
			Secondary outcomes:
			UTIs/resident: 0.04 (−0.01, 0.09)
		3. Feedback on prescribing & references to available guidelines; discussion of structural, organizational, social barriers to change.	All infections:
			antibiotics −0.12 (−0.23, -0.02)
			“wait & see” 0.143 (0.047, 0.240)
			Nitrofurantoin for lower UTI in women: - 0.077 (−0.247, 0.088)
Jump et al., 2012 [9] US	Pre/post; single site with dedicated physician/nurse practitioner care on 4 LTCF wards.	ID consultation service team (ID physician and nurse practitioner) once weekly on site and available by phone contact 24/7.	36 months pre compared with 18 months post: Reduction in
			→total antibiotics, 30.1%, p < 0.001
			→oral antibiotics, 31.6%, p,0.001
			→intravenous antibiotics, 25%, p = 0.001
			Positive *C. difficile*/1,000 days decreased: time series, p = 0.04

LTC: long term care facility; UTI: urinary tract infection.

The interventions described were relatively limited for two of the reports. One trial randomized 8 long term care facilities to intervention or control [7]. The 36 physicians providing care at the intervention homes were mailed a prescribing guide together with each individual physician’s prescribing profile for the previous three months; these physicians received a second mailing with the same information 4 months later. The control homes continued usual care, without any interventions. There was a significant improvement in antimicrobial prescribing reported for the intervention homes during the three months following the second mailing. By 15 months after the last mailing a trend to improvement for the intervention homes remained, but the difference was not statistically significant [7]. A second report described the impact of introducing an infectious diseases consultation service for long term care wards at one Veteran’s Affairs medical centre [9]. Consultation advice was available 24/7 by phone, with once weekly on site specialist case review by an infectious diseases physician and a nurse practitioner. Systemic antimicrobial use decreased by 30% in the 18 months following institution of the consultation service compared with the 36 months prior to implementation. A significant decrease in *C. difficile* colitis cases on the long term care wards was also observed.

The two other studies describing comprehensive programs implemented multimodal stewardship activities (Table 1). Schwartz [6] describes a program which incorporated small group educational sessions for twenty full-time, salaried internists who provided medical care to all residents on long term care wards at a single public hospital. These sessions introduced and discussed national guidelines for antimicrobial use in long term care facilities together with results of a local audit of infection management practices and local hospital resistance data. Compliance with the antimicrobial guidelines was evaluated through a chart review of antimicrobial use in a random sample of long term care patients prior to and following the intervention, with physician feedback of the results. Mean antimicrobial starts and monthly antimicrobial days fell significantly after the intervention. There were also significant improvements in antimicrobial use meeting guidelines during the 18 months of the intervention, and this was sustained during a two year post-intervention period. However, the study wards also had access to on site infectious diseases specialist consultation and the potential impact of this resource was not considered. Pettersson et al. [8] described an education intervention to improve antimicrobial use in a cluster randomized trial in Swedish long term care facilities. The interventions included educational small group sessions with facility nurses and physicians, guidelines adapted for the local context, written materials, and feedback on prescribing. The primary outcome was fluoroquinolone use for urinary infection. At the end of the 2 year intervention period fluoroquinolone use for urinary infection had decreased for both groups, with no difference between the intervention and control homes. There were, however, significant differences favouring the intervention homes in some secondary outcomes, including decrease in any antimicrobials given for all infections and increase in a “wait and see” approach of observation with delayed empiric antimicrobial prescription, if necessary.

Two reports describe programs addressing treatment of pneumonia in residents of long term care facilities (Table 2). Naughton [10] describes a randomized controlled trial undertaken in 10 nursing homes. A multifaceted education program and treatment guidelines were implemented for all facilities, with homes randomized to only physicians and nurse practitioners receiving the intervention or to a multidisciplinary intervention which also included education of nurses. The program included small group consensus meetings with physicians/nurse practitioners for development of guidelines and, for nurses, a one hour training session to acquaint them with the guidelines. Laminated pocket cards and laminated posters located by the telephone on the wards summarized these guidelines. There were no differences between the randomized training groups in antibiotic use for pneumonia prior to or following the intervention. In the pre/post analysis, a significant increase in guidelines compliance for use of parenteral compared with oral antibiotics was observed. When this outcome was stratified by the two randomized groups, improvement was only observed for the homes with the multidisciplinary intervention. There were no changes in mortality or hospitalization for pneumonia in any of the study homes. In the second report describing pneumonia treatment, a nonrandomized trial compared eight intervention and eight control homes during a 2-year observation period [11]. This program included optimized practices for immunization and laboratory tests, interactive education sessions for staff, support to facilitate institutional change, and academic detailing for physicians. The intervention was implemented by a multidisciplinary team of physicians, nurses and pharmacists. During the three study years, there were no differences in optimal antimicrobial use or duration of antibiotic use for episodes of pneumonia in the intervention compared to the control homes. A significant increase in antibiotics being initiated within 4 hours was observed for the intervention homes, but the relevance of this standard of practice for the nursing home setting is not known.

**Table 2:** Outcomes of antimicrobial stewardship programs focusing on a single infection in long term care facilities (LTCF)

**References, infection**	**Design**	**Interventions**	**Outcomes**
Pneumonia			
Naughton, 2001 [10] US	Randomized, controlled; 10 LTF	1. Small group consensus process for guideline development with physician/nurse practitioners.	1. No differences in antimicrobial use consistent with guidelines between two randomized groups.
	Facilities randomized to physician/nurse practitioner intervention only, or multidisciplinary (registered nurses/LPN’s).		
			2. In a pre/post analysis:
			a) Pre/post parenteral antibiotics meeting guidelines 50% vs 81.8% (p = 0.06) for multi-disciplinary group and 65% vs 69% (p = 0.73) for physician/practitioners.
		2. Nurses: 1 hour training session on guidelines.	
		3. Laminated pocket cards summarizing guidelines.	
			b) No change in 30 day mortality or hospitalization.
		4. Laminated posters with guidelines by telephone.	
Linnebur, 2011 [11] US	Non-randomized: 8 intervention homes, 8 control homes.	1. Optimized immunization, diagnostic testing at facility level.	1. Optimal antibiotic use pre/post: intervention 60% vs 66%; control 32% vs 39% (NS).
		2. Interactive educational sessions for NH staff to improve vaccination rates and nursing assessment skills.	
			2. Duration of antibiotics, no difference.
			3. Antibiotics within 4 hours: 57% → 75% vs 38% → 31% (p < 0.001)
		3. Study liaison nurse to facilitate change.	
		4. Academic detailing to physicians	
Urinary tract infection			
Loeb, 2005 [12] Canada	Cluster randomized: 24 NH	1. Diagnostic & treatment algorithm for urinary infection.	1. Antimicrobial courses for suspected urinary infection: 1.17 vs 1.59/1,000 resident days– difference - 0.49 (−0.93, -0.06)
		2. Small group interactive sessions for nurses using case scenarios - video-tapes of sessions, written material, continuing outreach visits.	
			2. Total antimicrobial use: 3.52 vs 3.93/1,000 days difference −0.37 (−1.17, 0.44)
		3. One on one interviews with physicians.	
		4. Pocket cards and posters with algorithms.	
Zabarsky, 2008 [13] US	Pre/post: single LTCF	1. Education of nursing staff to discourage urine cultures in absence of symptoms. Pocket cards with criteria for cultures.	In 6 months after intervention:
			1. Inappropriate urine cultures: 2.6 → 0.9/1000 (p < 0.04)
			2. Treatment of ASB: 167.1 → 117.4/1000 pt-days (p = 0.0017)
			3. Total antimicrobial days: 167.7 → 117.4/1,000 pt days (p < 0.001) Reductions maintained for 7 to 30 months while intervention continued.
		2. Education of physicians/nurse practitioners re current guidelines not to treat ASB and adverse effects of antibiotics. Pocket cards for diagnosis and treatment of symptomatic urinary infection.	
		3. Posters at computer stations used by nurses/primary care physicians.	
		4. Follow-up educational sessions semi-annually by infection control nurse with case based feedback of inappropriate practices.	
Rummukainen, 2012 [14] Finland	Pre/post; 25 primary care hospitals, 39 NH	1. Visit of team to facility with education: structured interview of individual patients, review of systemic antimicrobials, diagnostic practices for UTI.	Proportion of patients receiving antibiotic prophylaxis for UTI: 13% in 2005 → 6% in 2008 (p < 0.001)
		2. Regional guidelines developed and published.	
		3. Annual questionnaire to reinforce guideline consistent use of antibiotics.	

NH: nursing home, LTCF: long term care facility; ASB: asymptomatic bacteriuric.

Three studies describe stewardship programs which addressed specific aspects of management of urinary tract infection (Table 2). A cluster randomized trial evaluated the impact of implementation of consensus guidelines identifying minimum criteria which should be present prior to institution of empiric antimicrobial therapy for treatment of urinary infection. There were 12 nursing homes randomized to intervention and 12 to usual care [12]. Algorithms for diagnosis and treatment of urinary infection were developed to support use of the minimum criteria. The intervention program included nursing education in small group interactive sessions, video tapes and written material, outreach visits and one-on-one physician detailing. Over the 12 month study period there was a significant decrease in the number of antimicrobial days given for suspected urinary infection in the intervention compared with control homes, but no difference between the two groups in total antimicrobial days for all indications. The difference between intervention and control groups appeared to wane over time, despite visits to the homes by study staff every three months to address questions and audit antimicrobial use. An American study described a program which addressed the specific goal of discouraging inappropriate treatment of asymptomatic bacteriuria [13]. Interventions included education of nursing staff about appropriate collection of urine specimens and education of primary care physicians and nurses about recommendations that asymptomatic bacteriuria should not be treated. Follow-up was provided every six months and included direct individual feedback regarding specific cases identified with inappropriate urine cultures sent or treatment of asymptomatic bacteriuria given. In the six months following implementation there were significant decreases in the proportion of inappropriate urine specimens sent for culture, episodes of treatment of asymptomatic bacteriuria, and total antimicrobial days. These reductions were maintained during the following seven to 30 months, while the intervention was continued. The third study implemented programs in 39 nursing homes in Finland to specifically address the problem of widespread use of long-term prophylaxis for urinary infection identified in national prevalence surveys of antimicrobial use [14]. A team visited each of the facilities to customize guidelines for local use and to provide discussions around individual patients. Guidelines were reinforced in yearly questionnaires forwarded to the facilities. A pre/post analysis concluded 13% of antibiotics given in 2005 were for prophylaxis for urinary infection, and only 6% in 2008, a significant difference.

## Conclusions

This review identified a limited number of studies which evaluate the implementation and effectiveness of antimicrobial stewardship programs for long term care residents. The programs described in these reports are all unique. There is no standardization of program components, implementation strategies, or evaluation. Thus, the generalizability of any of the programs for more widespread use in other facilities is uncertain. Most of the studies included simultaneous implementation of several different program components as well as input from practitioners to tailor specific program components for local implementation. Several distinct activities were usually implemented simultaneously, so the efficacy and relative importance of any single program component is unknown. While it seems appropriate to customize some program content for specific facility or regional needs, there should also be a core set of elements identified which are recommended for all antimicrobial stewardship programs. It is also desireable to standardize how outcomes are measured and reported, to allow comparisons among different programs.

Most of the studies reviewed report at least some improvements in antimicrobial use following introduction of the stewardship interventions. However, the improvements in some outcomes seem relatively limited given the intensity of the programs described [8,10-12]. Larger and more consistent improvements were reported where there was engagement of specialist physicians, including internists and infectious diseases consultants [6,9]. However, the two studies describing this approach were implemented in long term care wards of acute care facilities where resident management and access to specialist care differ from that of free-standing long term care facilities. Strategies which have incorporated education, local guidelines and feedback addressing antimicrobial use may have been less effective [7,8].

The studies restricted to pneumonia both reported no impact of comprehensive programs including education, guidelines and algorithms on overall antimicrobial use for this indication [10,11]. The successful outcomes were for changes in use of oral compared with parenteral therapy [10] or initiating antimicrobials within 4 hours [11]. These outcomes are of uncertain relevance with respect to goals of stewardship to limit antimicrobial resistance or *C. difficile* colitis. The stewardship programs which addressed more limited goals relevant to urinary infection – treatment of asymptomatic bacteriuria or antimicrobial use for prophylaxis for urinary infection – were both reported to be effective [13,14]. However, a comprehensive approach for treatment of symptomatic urinary infection which was effective in decreasing antimicrobial use for this indication did not alter overall antimicrobial use in the study facilities [12]. Thus, focusing on limited goals where inappropriate use is an important problem may improve one aspect of antimicrobial use, but the diagnostic imprecision inherent in these facilities means any alterations should also be considered within the larger context of overall antimicrobial use.

None of these reports describe the impact of stewardship on the incidence or prevalence of antimicrobial resistance, an outcome of major current concern. In addition, for most of the studies reported, outcomes were evaluated only while the program remained active. Only one report described sustainability of changes in practice beyond the period of the intervention [6]. Finally, none of these reports describe the cost or cost-effectiveness of the programs. Given the resource limitations in these settings, sustainability and cost are both important considerations which should be addressed.

Current evidence is insufficient to support recommendations for a specific program, or any specific program components. Further evaluation of antimicrobial stewardship programs in long term care facilities, including the impacts of specific program components, is needed. Provision of on-site infectious diseases specialty consultation may be an effective intervention, but this is likely not realistic for most facilities. It seems reasonable for programs to have flexibility for customization to address local considerations. However, it should also be possible to standardize stewardship activities recommended for all facilities by identifying a library of effective program elements. Evaluation of the impact of stewardship programs on the relevant adverse outcomes of antimicrobial resistance and *C. difficile* colitis is also needed.

## Competing interests

The author declares that she has no competing interests.

## Authors’ contributions

The author is solely responsible for the inception, review, content, and writing of the manuscript.
